# Repair of complete bilateral cleft lip with severely protruding premaxilla performing a premaxillary setback and vomerine ostectomy in one stage surgery

**DOI:** 10.4317/medoral.20568

**Published:** 2015-06-02

**Authors:** Nabil Fakih-Gomez, Marta Sanchez-Sanchez, Fernando Iglesias-Martin, Alberto Garcia-Perla-Garcia, Rodolfo Belmonte-Caro, Luis-Miguel Gonzalez-Perez

**Affiliations:** 1 Department of Oral and Maxillofacial Surgery, Virgen del Rocio University Hospital, Seville, Spain

## Abstract

**Background:**

The authors present a technique for selected cases of CBCL. The primary repair of the CBCL with a severely protruding premaxilla in one stage surgery is very difficult, essentially because a good muscular apposition is difficult, forcing synchronously to do a premaxillary setback to facilitate subsequent bilateral lip repair and, thus, achieving satisfactory results. We achieve this by a reductive ostectomy on the vomero-premaxillary suture.

**Material and Methods:**

4 patients with CBCL and severely protruding premaxilla underwent premaxillary setback by vomerine ostectomy at the same time of lip repair in the past 24 months. The extent of premaxillary setback varied between 9 and 16 mm. The required amount of bone was removed anterior to the vomero-premaxillary suture. The authors did an additional simultaneous gingivoperiosteoplasty in all patients, achieving an enough stability of the premaxilla in its new position, to be able to close the alveolar gap bilaterally. The authors have examined the position of premaxilla and dental arch between 6 and 24 months. We did not do the primary nose correction, because this increased the risk of impairment of the already compromised vascularity of the philtrum and premaxilla.

**Results:**

The follow-up period ranged between 6 and 24 months. None of the patients had any major complication. During follow-up, the premaxilla was minimally mobile. We achieved a good lip repair in all cases: adequate muscle repair, symmetry of the lip, prolabium and Cupid’s bow, as well as good scars.

**Conclusions:**

To our knowledge, there are few reports of one stage surgery with vomerine ostectomy to repair CBCL with severely protruding premaxilla. Doing this vomerine ostectomy, we don’t know how it will affect the subsequent growth of the premaxila and restrict the natural maxillary growth. Applying this alternative treatment for children with CBCL and protruded premaxilla without any preoperative orthopedic, we can successfully perform, in a single-stage surgery, a good primary lip repair at our center. Further confirmations of this surgery with follow up and anthropometric studies of these patients during childhood and adolescence are required.

**Key words:** Protruding premaxilla, bilateral cleft lip, vomerine ostectomy, one stage surgery, Millard II technique.

## Introduction

Prominent premaxilla is a characteristic of infants with complete bilateral cleft lip. It is related to the attachment of premaxilla to the vomer and the nasal septum via the septo-premaxillary ligament in the absence of lateral strains. The protruding twisted premaxilla adds the problems of surgical management of bilateral complete cleft of lip patients (CBCL). Premaxilla is unrestrained by either of the maxillary alveoli and only attached to nasal septum by septomaxillary ligament. In normal children, the cartilaginous septum must slide forward in relation to the premaxillary region owing to the restraint on the premaxilla by the lip and lateral maxillary segments. In bilateral cleft, the premaxilla is carried forward at the same rate as that of the growing septum to which it is firmly held. The premaxilla has only one restraining connection, the vomer. This restrain is realized as a tension between these bones borne by the vomero-premaxillary suture, thus creating the condition for bone formation (1,2).

Various appliances for premaxillary setback such as extraoral head cap, elastic straps, tapes, Latham appliance and Burston plate have been successful after various surgeries and long time treatment (3-6). The literature has mentioned about the lip adhesion as the treatment of protruding premaxilla; it is not always successful to mold premaxilla without muscle repair. In addition, it adds to one more surgery and also adds scars to the lip making the next repair more difficult. The problems are compounded in BCLP by protruding premaxilla (7).

So conditioned on familiar demand and lack of preoperative orthopedic treatment force us to use one stage surgery to treat them. This primary repair of CBCL with a severely protruding premaxilla in one stage surgery is very difficult, essentially because a good muscular apposition is difficult, forcing synchronously to do a premaxillary setback to facilitate subsequent bilateral lip repair.

While following the principals described by Millard in 1977 (8,9), we added the vomerine ostectomy technique to suit the local circumstances. So by this reductive ostectomy on the vomero-premaxillary, we can perform it in one stage surgery. At the same time as premaxillary setback, the blood supply of the premaxilla is impaired, with the risk of premaxillary necrosis. There are few reports of one stage surgery with vomerine ostectomy to repair CBCL with severely protruding premaxilla. (10-13). To our knowledge, this is the first report using both combinations vomer ostectomy and Millard II technique.

## Patient and Methods

- Surgical Technique:

Under general anesthesia, supine position and minimal extension of the patient’s neck, the procedure begins with vomerine ostectomy. The required of bone was removed anterior to the vomero-premaxillary suture. We did additional simultaneous gingivoperiosteoplasty, achieving an enough stability of the premaxilla in its new position, to be able to close the alveolar gap bilaterally, followed by suturing the orbiculus muscle then using Millard II technique to repair the lip. We did not do primary nose correction, because this increased the risk of impair the already compromised vascularity of the philtrum and premaxilla. The alar cartilages are readjusted through rim incisions.

- Clinical Cases:

Between December 2011 and April 2013, 4 patients with complete bilateral cleft lip with protruding premaxila underwent premaxillary setback by vomerine ostectomy at the same time of lip repair. All patients had palatine cleft associated who they underwent a second time surgery to repair the palatine cleft. Patient ages ranged between 7 months and 4 years 5 months with a mean age of 19 months. There were 2 males and 2 females. None of the children had undergone any previous orthodontic treatment.

**Figure 1:**
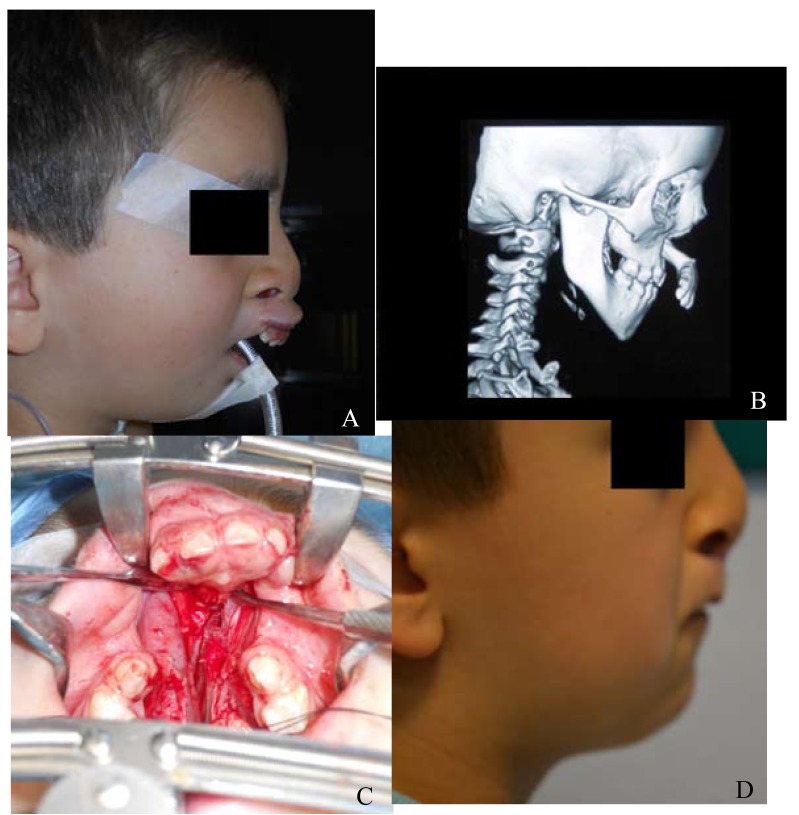
(case 1): A 4-year-old male with CBCL with protruding premaxilla and palate cleft. (A) Preoperative anterior and lateral view. (B) CT scan showing the extreme protrusion of the premaxilla with a vertical excess ; Notice the length of the vomerine bone needed to be excised on CT scan. (C) Occlusal intra-oral view, showing the unrestrained anterior nasal septal and vomero-premaxillary suture after wedge ostectomy of the vomer. (D) Postoperative results on lateral view (18 months postoperatively).

**Figure 2:**
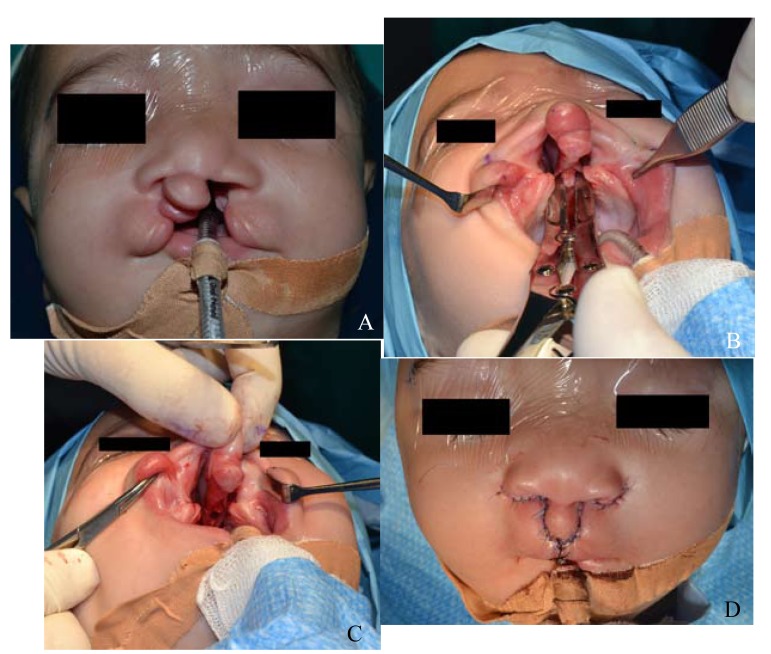
(case 2): A 6 months male with CBCL with protruding premaxilla and palate cleft. (A) Preoperative anterior view. (B) Occlusal intra-oral view, showing vomero-premaxillary suture and the site of the wedge ostectomy of the vomer with a bone cutter. (C) Occlusal intra-oral view, showing the gap after the withdrawal of the wedge osteotomized vomer. (D) Lip repair and immediate postoperative results on anterior view after suturing the orbicularis muscle and the gingivoperiosteoplasty.

**Figure 3:**
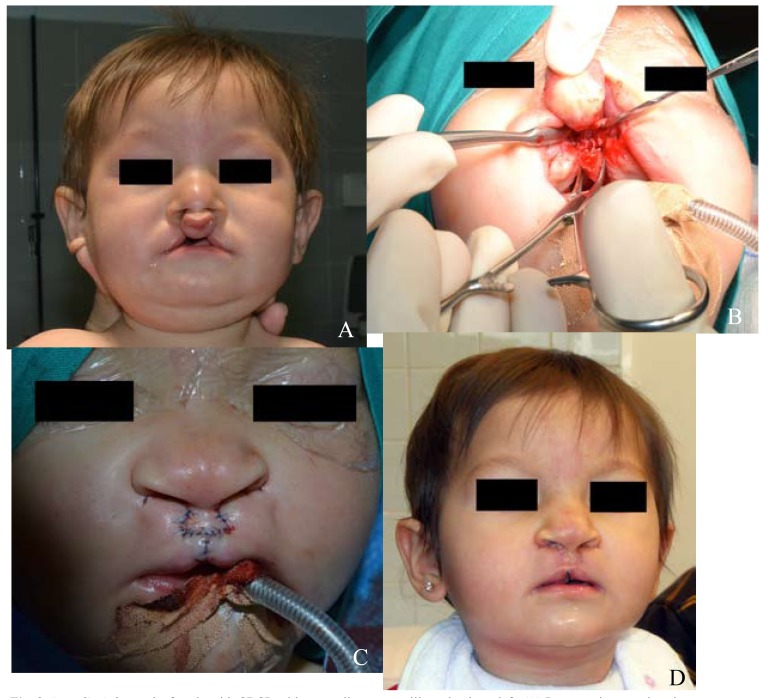
(case 3): A 9 months female with CBCL with protruding premaxilla and palate cleft. (A) Preoperative anterior view. (B) Occlusal intra-oral view, showing the wedged osteotomized vomer. (C) Lip repair and immediate postoperative results on anterior view. (D) Postoperative results on anterior view (1 month postoperatively).

**Figure 4:**
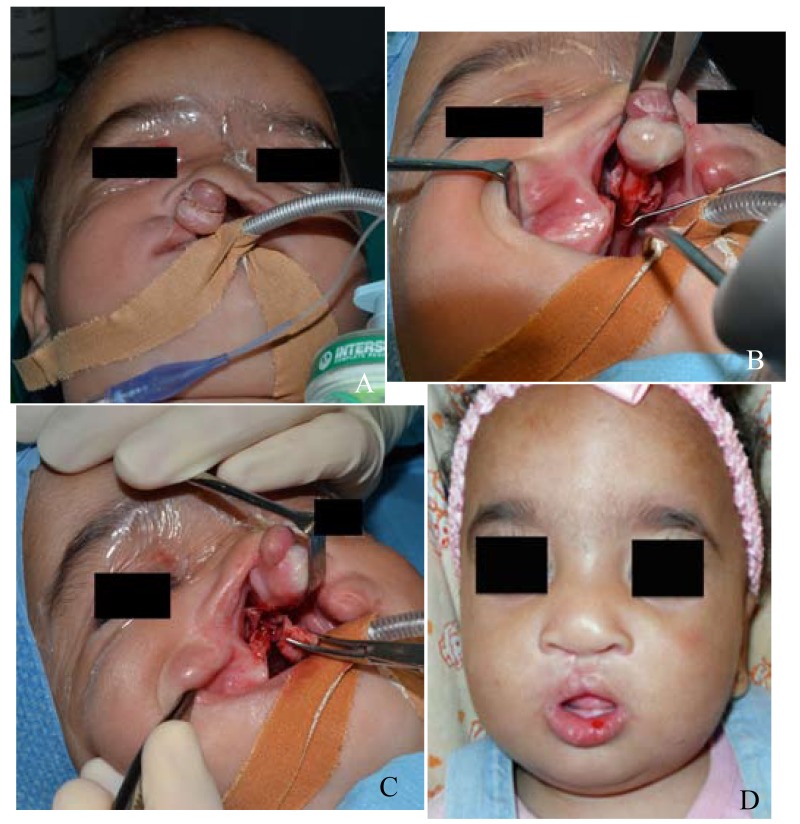
(case 4): A 7 months female with CBCL with protruding premaxilla and palate cleft. (A) Preoperative anterior view. (B) Occlusal intra-oral view, showing vomero-premaxillary suture and the site of the wedge ostectomy of the vomer. (C) Occlusal intra-oral view, showing the wedged osteotomized vomer. (D) Postoperative results on anterior view (1 month postoperatively).

- Case 1: A 4-year-old male patient with no family history nor risk factors, nor associated anomalies and syndrome, that after being treated several times previously in other centers, referred to our center to resolve the palate cleft and the projected pretreated premaxilla with the closure of the cleft lip. Only in this patient we repaired at the same time the palate cleft with the vomerine ostectomy followed by a new lip repair 4 months later. We underwent premaxillary setback at the time of palate repair because he had underwent other lip surgeries in other clinics without treating the projected premaxila (Fig. 1).

- Case 2: A male patient of 6 months with no family history nor risk factors, nor associated anomalies and syndrome (Fig. 2).

- Case 3: A female patient of 9 months with familiar history (paternal uncle) with cleft lip, with mother history of drug intake and no associated anomalies nor syndrome (Fig. 3).

- Case 4: A female patient of 7 months without family history or risk factors and bilateral hypoacusis (Fig. 4). The three other cases underwent the surgical technique described before. Case 2 had cleft palate repair 13 months later. The other two remaining cases are awaiting surgical intervention for palate cleft.

The extent of premaxillary setback varied between 9 and 16 mm. The required amount of bone was removed anterior to the vomero-premaxillary suture. Only in case 1, being older, elastic traction is performed to gradually retract the premaxilla. To achieving an enough stability of the premaxilla in its new position, and to be able to close the alveolar gap bilaterally, we did an additional simultaneous gingivoperiosteoplasty.

## Results

The follow-up period ranged between 6 and 24 months. We examined the dental arch and position of the premaxilla clinically and documented photographically in all revisions. None of the patients had any major complication, such as loss of the premaxilla for any ischemic episode or vascular compromise of the premaxilla or skin dehiscence of the lip. During follow-up, it was noted that the premaxilla was minimally mobile in all patients. The extent of premaxillary setback varied between 9 and 16 mm. We achieved a good lip repair in all cases with adequate muscle repair, with an excellent symmetry of the lip, prolabium and Cupid’s bow as well as good scars. No fistulas appearances were documented. Referring to case 1, speech evaluation showed improvement with regard to intelligibility, hypernasality, and articulation error. However, as expected, it was not normal because of obvious factors, such as late palate repair and habitual patterns. In the other cases, are still young to record intelligibility and two of them is still awaiting palate cleft repair.

## Discussion

It is best to initiate treatment as early as possible because surgical scars are less visible following surgery in infants (14). In addition, the bone and cartilage tissues are soft and moldable in infants facilating more the surgical correction, and normal oral function can be established once the lip structures have been repaired anatomically. Psychological problems for the baby and family are also minimized (15).

Protrusion of the premaxilla in CBCL can be seen as early as 10 weeks of gestation (1). Unrestrained anterior nasal septal and vomero-premaxillary suture growth, combined with lack of bony and soft tissue continuity, and disruption of balance between the circumoral musculature and the tongue are thought to produce the classic bilateral premaxillary deformity (16,17). This premaxillary relationship may result in significant functional problems such as absence of proper anterior occlusion, lateral mobility of the premaxillary segment and labial or palatal oronasal fistulae with consequent problems in speech and oral hygiene. Prominence or vertical overdevelopment of the premaxilla also may result in significant psychological harm during a child’s formative years. Bilateral cleft lip repair with protruding premaxila remains one of the most challenging problems for surgeons. To our knowledge, there are few reports of one stage surgery with vomerine ostectomy to repair CBCL with severely protruding premaxilla (10-13).

Correction of the displaced premaxilla by orthodontic treatment alone is impossible. On the other hand, surgical repositioning is technically demanding and may interfere with the blood supply of the premaxilla. Two-stage procedure was advised by some authors (18), but osteotomy of the premaxilla, in combination with the correction of the lip cleft, has been also reportedly successful in selected cases, especially en older patients. This procedure enables closure of large fistulae (increasing the chances of success of lip reconstruction without tension in the lip skin), facilitates overjet and overbite correction, and may have psycho social advantages for the child and his family which demand one time surgery and where we can not offer them nasoalveolar moldings (NAM) or previous orthodontic treatment. Although early results with preoperative NAM have good result, objective analysis of long-term outcome has not yet been reported (19,20).

Multiple studies compared unrepaired adult cleft lip and cleft palate maxillary morphology to that in adults with repaired clefts (21-26). The results are well- documented: adults with unrepaired cleft lips or palates have maxillas with a normal to slightly prognathic cephalometric position anterior-posteriorly, and a normal to widened maxillary transverse width. Based on these studies, it is evident that the facial characteristics of an unrepaired adult cleft lip and palate patient are unique: a normal to slightly protrusive upper jaw, protruded maxillary anterior dentition, and a normal or slightly hypoplastic mandibular relationship. In addition, the cleft lip and cleft palate dimensions are that much greater, because the cleft grows proportionately with the surrounding anatomy. Padwa *et al*. (27) suggested that a protrusive premaxilla could be surgically repositioned after 6 to 8 years without deleterious effects on midfacial growth. Freihofer *et al*. (28) also noted that by this age (8 to 13 years), the development of the maxilla is far advanced and the growth disturbance at this age by premaxillary setback has only a relatively restricted negative influence. However, Friede and Pruzansky (29) noted that the majority of patients with bilateral complete cleft lip and palate who have undergone premaxillary setback have concave faces because of maxillary retrusion. Although this is a real sideffect, it is essential to bring the premaxillary setback in alignment with the lateral segment so that subsequent alveolar bone grafting and orthognathic procedures can be performed. Midfacial growth is easily documented by lateral cephalometry and by analysis of plaster dental casts. Many dental specialists emphasize that the degree of maxillary retrusion is a reflection of the surgical protocol or the operative technique. Some specialists believe that closure of the secondary palate should be delayed until childhood, despite studies to show that this strategy can result in poor speech. It has been clearly documented that adults with unrepaired cleft lip/palate exhibit normal facial growth (30,31). Repair of bilateral cleft lip/palate inhibits maxillary growth, yet the debate continues whether labial or palatal closures, or both, are responsible. Also Berkowitz *et al*. (32) has reported that early preoperative orthopedics leading buccal bite of the premaxilla with concave facial profiles worsen with time.

With limited information in the literature about follow-up studies of CBCL without any preoperative orthopedics, this study showed the outcome position of the premaxilla and the lip repair results. In all our four patients, applying this alternative treatment for children with CBCL and protruded premaxilla without any preoperative orthopedic, we can successfully perform, in a single-stage surgery, a good primary lip repair at our center. Single-stage primary operation is relatively easier to perform, as there are no scarred tissues. The vomerine ostectomy facilitate subsequent bilateral lip repair and adequate muscular repair, which is vital for labial function. Simultaneous gingivoperiosteoplasty on both sides provides better stability and alignment of the arches in addition to the bilateral closure of the alveolar gap. Using Millad II technique achieves an excellent symmetry of the lip, prolabium and Cupid’s bow as well as good scars. No complications were documented. Doing this vomerine ostectomy, we don’t know how it will affect the subsequent growth of the premaxila and restrict the natural maxillary growth, but as described before, it is essential to bring the premaxillary setback in alignment with the lateral segment so that subsequent alveolar bone grafting and orthognathic procedures if required can be performed. In addition many adolescents with repaired bilateral cleft lip need maxillary advancement to improve projection of the nasal tip, to protrude the upper lip, and to attain normal sagital skeletal harmony. However, we are supporters of performing early surgery, assuming the sequels to be corrected later but favoring the better development of language and aesthetics during childhood (14,15,29).

## Conclusions

Applying this alternative treatment for children with CBCL and protruded premaxilla without any preoperative orthopedic, we can successfully perform, in a single-stage surgery, a good primary lip repair at our center. Further confirmations of this surgery with follow up and anthropometric studies of these patients during childhood and adolescence are required.
